# Assessment of reverse remodeling predicted by myocardial deformation on tissue tracking in patients with severe aortic stenosis: a cardiovascular magnetic resonance imaging study

**DOI:** 10.1186/s12968-017-0392-0

**Published:** 2017-10-23

**Authors:** Ji-won Hwang, Sung Mok Kim, Sung-Ji Park, Eun Jeong Cho, Eun Kyoung Kim, Sung-A Chang, Sang-Chol Lee, Yeon Hyeon Choe, Seung Woo Park

**Affiliations:** 1 Division of Cardiology, Department of Medicine, Ilsan Paik Hospital, Inje University School of Medicine, Goyang, 10380 South Korea; 2 Division of Cardiology, Department of Medicine, Samsung Medical Center, Sungkyunkwan University School of Medicine, 81 Irwon-ro, Gangnam-gu, Seoul, 06351 South Korea; 3 Department of Radiology, Samsung Medical Center, Sungkyunkwan University School of Medicine, 81 Irwon-ro, Gangnam-gu, Seoul, 06351 South Korea; 4 Cardiovascular Imaging Center, Heart Vascular Stroke Institute, Samsung Medical Center, Sungkyunkwan University School of Medicine, 81 Irwon-ro, Gangnam-gu, Seoul, 06351 South Korea; 5 Division of Cardiology, Department of Medicine, National Cancer Center, Goyang, 10408 South Korea

**Keywords:** Aortic stenosis, Reverse remodeling, Myocardial fibrosis, Feature tracking, Strain, Myocardial strain

## Abstract

**Background:**

The technique of tissue tracking with balanced steady-state free precession cine sequences was introduced, and allowed myocardial strain to be derived directly, offering advantages over traditional myocardial tagging. The aim of this study was to evaluate the correlation between reverse remodeling as an outcome and left ventricular strain using cardiovascular magnetic resonance imaging (CMR) tissue tracking, and to evaluate prediction of reverse remodeling by myocardial deformation in patients with severe aortic stenosis (AS).

**Methods:**

We enrolled 63 patients with severe AS and normal left ventricular (LV) systolic function (ejection fraction > 60%), who underwent both CMR and transthoracic echocardiography (Echo) before surgical aortic valve replacement (AVR). CMR at 1.5 T, including non and post-contrast T1 mapping for extracellular volume (ECV), was carried out to define the amount of myocardial fibrosis. Cardiac Performance Analysis software was used to derive myocardial deformation as strain parameters from three short-axis cine views (basal, mid and apical levels) and apical 2, 3, and 4 chamber views. The primary outcome was reverse remodeling, as evaluated by regression of left ventricular mass index (LVMI).

**Results:**

Median follow-up was 28.8 months (interquartile range 11.3–38.3 months). As evaluated by LVMI between baseline and follow-up, mass regression was significantly improved after AVR (baseline 145.9 ± 37.0 [g/m^2^] vs. follow-up 97.7 ± 22.2[g/m^2^], *p* < 0.001). Statistically significant Pearson’s correlations with LVMI regression were observed for longitudinal global strain (*r* = −0.461, *p* < 0.001), radial strain (*r* = 0.391, *p* = 0.002), and circumferential strain (*r* = −0.334, *p* = 0.009). A simple linear regression analysis showed that all strain parameters could predict the amount of LVMI regression (*P* < 0.05), as well as non-contrast T1 value (beta = −0.314, *p* < 0.001) and ECV (beta = −2.546, *p* = 0.038). However, ECV had the lowest predictive power (multiple r^2^ = 0.071). Multiple regression analysis showed strain could independently predict the amount of LVMI regression and the longitudinal global strain (beta = −3.335, *p* < 0.001).

**Conclusion:**

Longitudinal global strain measured by CMR tissue tracking as a technique was correlated with reverse remodeling as LVMI regression and was predictive of this outcome. As a simple and practical method, tissue tracking is promising to assess strain and predict reverse remodeling in severe AS, especially in patients with suboptimal Echo image quality.

**Electronic supplementary material:**

The online version of this article (10.1186/s12968-017-0392-0) contains supplementary material, which is available to authorized users.

## Background

Patients with severe aortic stenosis (AS) are known to develop myocardial fibrosis. In AS patients, left ventricular (LV) hypertrophy and interstitial myocardial fibrosis are known sequelae of chronic pressure overload [1, 2]. Severe AS causes a pressure overloaded LV to compensate by altering its wall geometry in order to maintain wall stress [3, 4]. This hypertrophic remodeling process is pathological, with myocyte degeneration and replacement myocardial fibrosis, leading to ventricular dysfunction. Aortic valve replacement (AVR) removes aorto-valvular impedance, resulting in geometric changes (mass regression, volume reduction, and improved function) known as ‘reverse remodeling’ [5–7].

Alterations of myocardial texture resulting from AS such as myocardial fibrosis are hard to evaluate in clinical practice as there is no imaging tool to easily evaluate fibrotic changes. Non-contrast T1 values measured using cardiovascular magnetic resonance (CMR) have been correlated with diffuse myocardial fibrosis burden compared with histology in AS patients [8]. In addition, myocardial fibrosis was linked directly to AS prognosis [9], and can be measured with CMR imaging using late gadolinium enhancement (LGE) methods [10].

Myocardial strain analysis has shown to be superior to wall motion analysis to detect differences in myocardial deformation and to determine contraction timing. Myocardial deformation analysis as LV strain has been used for analysis of myocardial viability and myocardial fibrosis in various cardiac conditions [11–13]. Typically, AS causes LV pressure overload leading to LV hypertrophy, which is the basis for delayed and incomplete LV relaxation. These features of diastolic dysfunction can be quantified with CMR myocardial tagging, which has demonstrated abnormal strain and rotation values in prior studies [14–16].

CMR myocardial tissue tracking on balanced steady-state free precession (bSSFP) cine-imaging has been developed in order to satisfy the needs for fast and quantitative assessment of myocardial segmental and global strain analysis [17, 18]. CMR tissue tracking is the recently developed CMR-equivalent of speckle-tracking echocardiography [17]. CMR tissue tracking has been validated against myocardial tagging [19–21]. Importantly, CMR tissue tracking can be undertaken using bSSFP imaging, which is part of a routine CMR scan, and no additional sequences are required [22].

There are few data on the potential of CMR tissue tracking to define myocardial fibrosis and reverse remodeling in patients with severe AS. We hypothesized that the greater myocardial fibrosis is developed, the lower the degree of reverse LV remodeling. We evaluated the comparison of myocardial fibrosis using non-contrast T1 value and LV strain obtained by CMR tissue tracking. The final aims of this study were to evaluate the correlation between reverse remodeling and LV strain with a CMR tissue tracking technique and to predict reverse remodeling by myocardial deformation in patients with severe AS.

## Methods

### Study population and clinical outcome

The study population comprised 63 patients with severe AS and normal LV systolic function (ejection fraction > 60%) treated with surgical AVR. Patients were screened for inclusion in this study if they had been diagnosed with severe AS and were scheduled to undergo elective AVR between January 2012 and June 2015. Severe AS was defined as aortic valve area (AVA) less than 1 cm^2^ based on recommendations of the American Society of Echocardiography [23]. A total of 63 severe AS patients who underwent both transthoracic echocardiography (Echo) and CMR were enrolled. Patients with a glomerular filtration rate < 30 mL/min and highly impaired health status that made CMR examination impossible (severe chronic pulmonary disease, cardiac decompensation) were excluded, as were patients who met the classical contraindication for CMR (e.g., pacemaker, defibrillator, or claustrophobia).

A large number of self-reported healthy individuals undergo medical evaluation including CMR in our Health Promotion Center. From this large registry, we retrospectively selected the ten healthy individuals to serve as a control group for matching age and sex with case group. Informed consent was waived.

Echo and CMR were performed following a common standard protocol at baseline (within a week before surgery), and Echo was also performed at annual follow-up visits. Median follow-up was 28.8 months (interquartile range 11.3–38.4 months).

The end point was LV mass regression defined as the difference in LV mass index (LVMI) on preoperative Echo and the last available examination [24, 25]. The Institutional Review Board of Samsung Medical Center approved this study and all subjects gave written informed consent before the investigation.

### Cardiovascular imaging – Echo and CMR

#### Transthoracic echocardiography (Echo)

Conventional two-dimensional Echo was performed using commercially available equipment. LV dimension and other Echo parameters were obtained according to the guidelines of the American Society of Echocardiography [26]. LV end-diastolic and end-systolic volumes were measured from apical two- and four-chamber views, and LV ejection fraction (EF) was calculated using Simpson’s rule [26]. LV mass was calculated using the formula proposed by Devereux et al. [27] and corrected by body surface area to derive LVMI. AVA was calculated by the continuity equation, and the maximum pressure gradient across the restrictive orifice was estimated by the modified Bernoulli equation. Mean pressure gradient was calculated by averaging instantaneous gradients over the ejection period on the continuous-wave Doppler recordings [23]. All study populations as well as all Echo data were re-analyzed in a blinded fashion by two experienced sonographers (RDCS), with more than 10 years’ experience or >1000 cases, and who re-evaluated LVMI by Echo in all studies.

#### CMR protocol – Imaging acquisition

All patients underwent CMR at 1.5-T (Magnetom Avanto, Syngo MR B17 version; Siemens Medical Solutions, Erlangen, Germany) with a 32-channel phased-array receiver coil. CMR scans consisted of localizing images (axial, coronal, and sagittal), cine scans, pre T1 mapping, perfusion scans (both stress/rest scans, with an intravenous infusion of 0.1 mmol/kg gadobutrol at an injection rate of 3 mL/s, followed by a 30 mL saline flush), LGE scans, and post-contrast T1 mapping. All examinations were carried out by experienced technicians and supervised by an experienced radiologist.

After localization, cine images of the LV were acquired using a bSSFP sequence on 4-, 3-, and 2-chamber and short axis (SA) views to obtain contiguous slices that included the entire LV with a 6-mm slice thickness and 4-mm intersection gaps. At each level, cine images were composed of 30 phases per cardiac cycle. Cine images were obtained with the generalized autocalibrating partially parallel acquisitions (GRAPPA; Siemens Medical Solutions) reconstruction algorithm during multiple breath-holds. Cine images were acquired using retrospective electrocardiogram-gating with the following parameters: repetition time/echo time, 3.31 msec/1.31 msec; flip angle, 72°; field of view, 240 × 300 mm^2^; matrix, 256 × 150, GRAPPA acceleration factor, 2.

T1 mapping images that were acquired in short-axis. A short-axis section at the base level was acquired using modified Look-Locker inversion-recovery (MOLLI). Pre-contrast MOLLI was composed of 5 images in the first Look-Locker segment and 3 images in the second segment (“5–3” protocol). Finally, 8 images acquired during 11 heartbeats were obtained, and in-line motion correction and map generation were performed. Post-contrast MOLLI was composed of 4 images in the first Look-Locker segment, 3 images in the second segment, and 2 images in the third segment (“4–3-2” protocol). Finally, 9 images acquired during 11 heartbeats were obtained, and in-line motion correction and map generation were performed. The following readout parameters were used: section thickness, 8 mm; flip angle, 35; field of view (FOV), 360 × 307; effective TI (TIeff), 120 msec; TIeff. Increment, 80 msec; voxel size, 1.87 × 1.88 × 8 mm; TR/TE, 2.4/1.01 ms; partial Fourier, 7/8; and parallel imaging factor, 2. Post-contrast images were produced at the same positions within 15 min after the contrast injection.

#### Cardiovascular magnetic resonance myocardial tissue tracking analysis

CMR tissue tracking analyses were performed using commercially available software (cvi42 version 5, Circle Cardiovascular Imaging Inc., Calgary, Alberta, Canada). Two-, three-, and four-chamber and short axis images were uploaded into the software, which reconstructs a 3D model that is used for analyses of 2D- and 3D radial, circumferential and longitudinal LV strain. The preferred images were loaded into the analysis/viewer frame of the software and analyzed in a random order by two investigators (SMK with 10 years and JWH with 3 years of CMR) who were independently blinded to the clinical findings. Tissue tracking analysis was manually performed by drawing the endo- and epicardial surface in the end-diastolic phase (reference phase) using short axis stacked slices. A short axis reference point was manually delineated at the right ventricle (RV) upper and lower septal insertion of the LV for regional and global analysis of strain and the generation of polar map views. Next the software automatically drew up the contour and traced its myocardium voxel points throughout the remainder of the cardiac cycle. The algorithm determined and depicted the left borders of the LV myocardium in the following phases during a cardiac cycle based on the endo- and epicardial contours of reference phase. Analyses of strain were performed automatically in all slices by the software in 2D as well as 3D. We used the 17-segment model for assessments of regional and global myocardial mechanics [28].

Horizontal long-axis cines were tracked to derive longitudinal global strain, while short-axis cines were used to derive radial and circumferential strain. Only one measure of strain was calculated in the radial direction, as this direction (myocardial thickening and thinning) is perpendicular to the endocardial and epicardial borders, so both contours are required to derive transmural radial strain.

Contours for tissue tracking were determined by one investigator, and tissue tracking analysis was repeated in all subjects by another independent investigator, a radiology specialist.

### Statistical analyses

Continuous variables were compared using the Student’s *t*-test or Wilcoxon rank-sum test where applicable and are presented as mean ± standard deviations or medians with interquartile ranges (IQR). Categorical data were tested using Fisher’s exact test or the Chi-square test as appropriate. Correlations between primary outcome and strain parameters measured by CMR tissue tracking were assessed using Pearson’s method. For assessing fibrosis correlations, we also analyzed its correlation with strain parameters and non-contrast T1 value or extracellular volume (ECV).

To make a prediction on the change in LVMI regression based on any given strain value obtained with CMR tissue tracking, linear regression analysis was used. Only two cases as outliers were eliminated by Tukey’s robust outlier detection method. We also analyzed the additive value of strain parameters by CMR tissue tracking on non-contrast T1 and ECV for predicting reverse remodeling. All strain parameters (7 variables) were calculated with a stepwise multiple linear regression, and several multiple linear regression models were performed with strain parameters or baseline parameters on the following matrix of variables adjusted for age, gender, E velocity, e` velocity, max velocity of aortic valve (AV) and EF by Simpson’s method.

We calculated the inter and intra-observer interclass correlation coefficient (ICC) for LVMI by Echo. We also performed correlations between LVMI values acquired from Echo and CMR images at baseline by ICC and Bland-Altman plot.

All analyses were conducted using R software for Windows (version 3.3.2). *P*-values <0.05 were considered statistically significant.

## Results

### Baseline clinical and imaging parameters

A total of 63 patients (29 males) with severe AS who underwent surgery for AVR were included in the study. The mean age of all patients was 67.0 ± 8.5 years. The baseline characteristics of the study population are reported in Table 1. Mean LV EF of the study population was normal (60.8 ± 7.3%), and the median and interquartile range for LVMI was 137.8(117.4–166.5 [g/m^2^]). Additional Echo parameters and AV flow measurements are listed in Table 1.

**Table 1:** Clinical characteristics and echocardiographic parameter of the study population

Age, years	67 (60–74)
Male gender	29 (46.0%)
Systolic blood pressure (mmHg)	121 (113–137)
Diastolic blood pressure (mmHg)	69 (61–75)
Heart rate (bpm)	69 (62–78)
Past medical history	
Atrial fibrillation	3 (4.8%)
Diabetes mellitus	13 (20.6%)
Hypertension	30 (47.6%)
Hyperlipidemia	17 (27.0%)
Ex-smoker	6 (9.5%)
Current smoker	4 (6.3%)
Creatinine (mg/dL)	0.83 (0.71–1.02)
Diameters of aortic root and ascending aorta	
Diameter of aortic annulus (mm)	21 (20–23)
Diameter of Sinus of Valvsalva (mm)	33.6 (29.6–37.0)
Diameter of sinotubular junction (mm)	27.8 (24.9–30.9)
Diameter of ascending aorta (mm)	38.4 (34.6–42.8)
Baseline echocardiographic parameters	
LVEF (%)	61 (56–66)
LVEDV(mL)	129.1 (94–154.2)
LVESV (mL)	48.9 (36–64)
LVEDD (mm)	51 (48–55)
LVESD (mm)	30 (27–36)
Interventricular septum (mm)	12 (10–13)
Left ventricular posterior wall (mm)	11 (11–12)
Left atrium size (mm)	42 (39–46)
LAVI (mL/m2)	43.6 (37.3–54.9)
LVMI (g/m2)	137.8 (117.4–166.5)
E velocity (m/s)	0.69 (0.53–1.04)
Deceleration time (msec)	264 (209–322)
e’ velocity (m/s)	0.05 (0.04–0.06)
E/e’ ratio	15.06 (10.63–20.51)
Parameters of grade of aortic stenosis	
Max velocity of AV(m/s)	5.26 (4.56–5.62)
AV velocity time integral (cm)	123.7 (106.6–146.7)
Mean pressure gradient of AV (mmHg)	61.7 (50.1–78.9)
LVOT velocity time integral (cm)	24.8 (21.6–29.7)
AVA (cm2)	0.72 (0.57–0.83)
AVAI (cm2/m2)	0.44 (0.38–0.51)

Data are presented are number of patients (percent) or median (interquartile range)

*LVEF* left ventricular ejection fraction, *LVEDV* left ventricular end-diastolic volume, *LVESV* left ventricular end-systolic volume, *LVEDD* left ventricular end-diastolic dimension, *LVESV* left ventricular end-systolic dimension, *LAVI* left atrium volume index, *LVMI* left ventricular mass index, *AV* aortic valve, *LVOT* left ventricular outflow tract, *AVA* aortic valve area, *AVAI* aortic valve area (indexed)

The LVMI measured by Echo inter-observer ICC was 0.85 (95% confidence interval: 0.79–0.89, *p* < 0.001) and the intra-observer ICC was 0.86 (95% confidence interval: 0.81–0.90, *p* < 0.001). In addition, there was a positive correlation between LVMI values of the baseline Echo and CMR images (*r* = 0.73, *p* < 0.001), and ICC was 0.723 (95% confidence interval: 0.580–0.823, p < 0.001). We also presented the Bland-Altman plot with LVMI values of the baseline Echo and CMR (Additional file 1: Figure S1). The median time duration from AVR to follow-up Echo was 833 days (interquartile range 372–1183 days) (Additional file 1: Figure S2).

### Correlation of non-contrast T1 value, extracellular volume, and strain parameters by CMR tissue tracking

We selected 10 healthy individuals of similar age and gender to compare with the 10 patients (the study population with severe AS) on non-contrast T1 value and ECV. The fibrosis quantification as measured by CMR was significantly different between patients with severe AS and healthy controls in terms of non-contrast T1 value and ECV (non-contrast T1 value [1024.4 ± 36.7 vs. 968.3 ± 52.0, *p* = 0.013] and ECV [28.0± 2.6 vs. 23.5 ± 1.3, *p* < 0.001]). The T1 value of healthy individuals was consistent with previous studies, as 1029.4 ± 56.8 by Nacif et al. [29], and 1025 ± 41 by Roujol et al. [30].

**Fig. 1:**
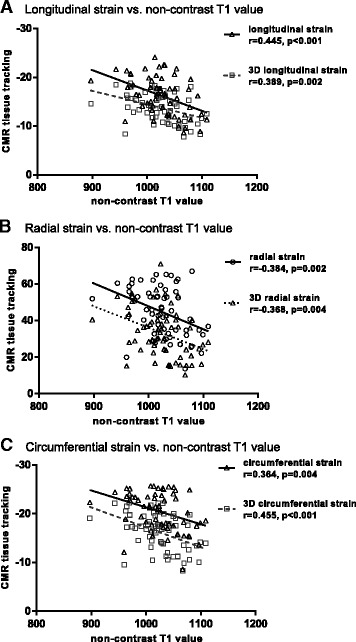
Correlation between non-contrast T1 value and strain measure by cardiovascular magnetic resonance (CMR) tissue tracking. Various kinds of strain including longitudinal strain, 3D longitudinal strain, radial strain, 3D radial strain, circumferential strain, and 3D circumferential strain by CMR were compared with non-contrast T1 values

There was a negative correlation between the amount of myocardial fibrosis determined by non-contrast T1 value and longitudinal global strain (*r* = 0.445, *p* < 0.001) and 3D longitudinal global strain (*r* = 0.389, *p* = 0.002) by CMR tissue tracking (Fig. 1a). There was a significant negative correlation between non-contrast T1 value and radial strain (*r* = −0.384, p = 0.002) and 3D radial strain (*r* = −0.368, *p* = 0.004) (Fig. 1b). Additionally, there was also a negative correlation between non-contrast T1 value and circumferential strain (*r* = 0.364, *p* = 0.004) and 3D circumferential strain (*r* = 0.455, *p* < 0.001) (Fig. 1c).

**Fig. 2:**
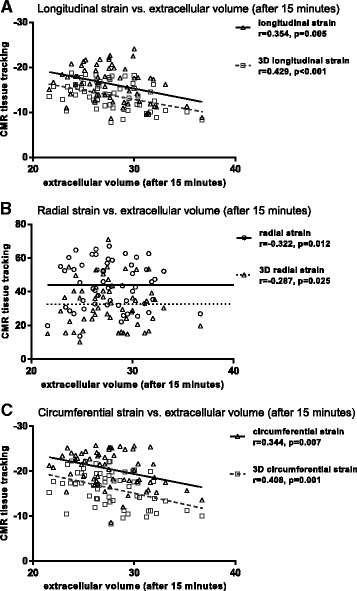
Correlation between the value of extracellular volume (ECV) and strain measure by CMR tissue tracking. Various kinds of strain including longitudinal strain, 3D longitudinal strain, radial strain, 3D radial strain, circumferential strain, and 3D circumferential strain by cardiac magnetic resonance were compared with ECV

ECV showed a similar correlation pattern with strain by tissue tracking as a non-contrast T1 value. There was a negative correlation between ECV and longitudinal global strain (*r* = 0.354, *p* = 0.005) and 3D longitudinal global strain (*r* = 0.429, *p* < 0.001) by CMR-tissue tracking. There was a significant negative correlation between ECV and radial strain (*r* = −0.322, *p* = 0.012) and 3D radial strain (*r* = −0.287, *p* = 0.025). Additionally, there was also a negative correlation between ECV and circumferential strain (*r* = 0.344, *p* = 0.007) and 3D circumferential strain (*r* = 0.408, *p* = 0.001) (Fig. 2c).

### Left ventricle reverse remodeling and strain by CMR tissue tracking

**Fig. 3:**
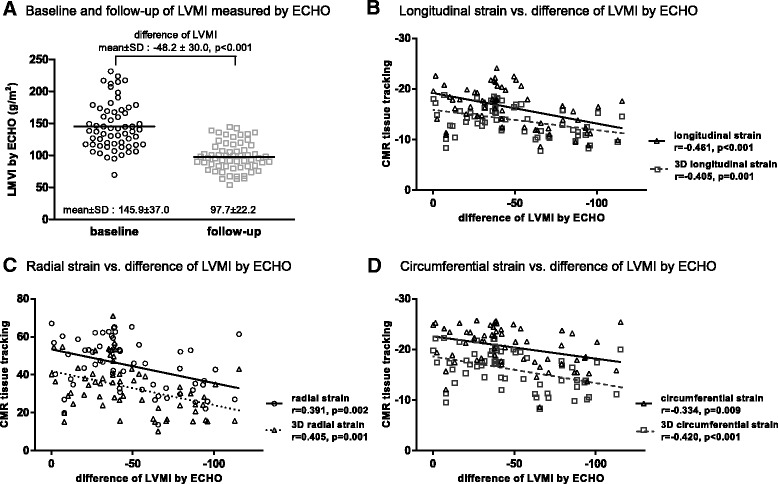
Correlation between left ventricle (LV) reverse remodeling by transthoracic echocardiography (Echo) and strain by CMR tissue tracking. This was compared using left ventricular mass index (LVMI) between baseline and follow-up by Echo. Correlation between reverse remodeling as LVMI difference between baseline and follow-up Echo and respective strain measured by CMR tissue tracking. (SD; standard deviation, LVMI; left ventricular mass index)

The result of baseline and follow-up Echo are shown in Fig. 3a. LVMI was 145.9 ± 37.0 (g/m^2^) on baseline study of Echo and 97.7 ± 22.2 (g/m^2^) on follow-up study. Additionally, as follow-up there was a significant LVMI reduction (the difference of LVMI: −48.2 ± 30.0 [g/m^2^], *p* < 0.001).

Significant Pearson’s correlations were seen between reverse remodeling defined as LVMI difference between baseline and follow-up Echo, and longitudinal global strain (*r* = −0.461, *p* < 0.001), radial strain (*r* = 0.391, *p* = 0.002) and circumferential strain (*r* = −0.334, *p* = 0.009). All types of 3D strain were also correlated with reverse remodeling, including 3D longitudinal global strain (*r* = −0.405, *p* = 0.001), 3D radial strain (*r* = 0.405, *p* = 0.001) and 3D circumferential strain (*r* = −0.420, *p* < 0.001) (Fig. 3).

To perform linear regression analysis, outliers were eliminated, and finally 61 cases were analyzed. Simple linear regression showed strain parameters measured by CMR tissue tracking could predict LVMI regression, in terms of longitudinal global strain (beta = −3.533, *p* < 0.001), radial strain (beta = 0.860, *p* = 0.002), and circumferential strain (beta = −3.360, *p* < 0.001). The all types category for other types of 3D strain also predicted the outcome, as well as 3D longitudinal global strain (beta = −4.107, *p* = 0.001), 3D radial strain (beta = 0.914, *p* = 0.001), and 3D circumferential strain (beta = −3.360, *p* < 0.001). In terms of results, the degree of reverse remodeling prediction was similar between 2D and 3D deformation parameters.

The non-contrast T1 value (beta = −0.314, *p* < 0.001) and ECV (beta = −2.55, *p* = 0.038) predicted outcomes. However, ECV had the lowest predictive power (multiple r^2^ = 0.071). We also analyzed the additive value of all strain parameters based on non-contrast T1 and ECV. After adding the strain parameters measure by CMR tissue tracking respectively, its predictive powers were significantly increased (Table 2). Additionally, we also performed an analysis on the presence of late gadolinium enhancement and reverse remodeling, but it was not significant in a linear regression (beta = 8.327, *p* = 0.17).

**Table 2:** Simple linear regression analysis of variables for the prediction of left ventricular reverse remodeling

	Multiple r^2^	Adjusted r^2^	Estimated beta	Standard error	p-value
longitudinal global strain	0.213	0.199	−3.533	0.885	<0.001
3D longitudinal global strain	0.164	0.15	−4.107	1.206	0.001
radial strain	0.153	0.138	0.86	0.264	0.002
3D radial strain	0.164	0.15	0.914	0.268	0.001
circumferential strain	0.112	0.097	−2.532	0.93	0.009
3D circumferential strain	0.177	0.163	−3.36	0.945	<0.001
non-contrast T1 value	0.191	0.177	−0.314	0.084	<0.001
extracellular volume after 15 min	0.071	0.055	−2.546	1.201	0.038
Additive value of strain parameters					
non-contrast T1 value					
longitudinal global strain	0.280	0.255	−2.549	0.954	0.01
3D longitudinal global strain	0.256	0.23	−2.81	1.245	0.028
radial strain	0.249	0.223	0.575	0.271	0.038
3D radial strain	0.260	0.234	0.638	0.274	0.023
circumferential strain	0.226	0.199	−1.529	0.94	0.11
3D circumferential strain	0.253	0.227	−2.234	1.019	0.032
extracellular volume after 15 min					
longitudinal global strain	0.225	0.198	−3.214	0.947	0.001
3D longitudinal global strain	0.175	0.146	−3.616	1.338	0.009
radial strain	0.175	0.146	0.749	0.277	0.009
3D radial strain	0.189	0.161	0.808	0.278	0.005
circumferential strain	0.138	0.108	−2.085	0.984	0.038
3D circumferential strain	0.187	0.159	−2.99	1.037	0.006

All strain parameters were measured by cardiovascular magnetic resonance myocardial tissue tracking

Initially, 3D circumferential strain was only selected in an adjusted stepwise multiple regression analysis of 6 strain parameters, for analysis of main effects. Then, we created two models: Model 1, the stepwise selection was adjusted with interaction effects in mind; Model 2, included the main effect term with adjustments for baseline parameters including age, gender, E velocity, e` velocity, max velocity of AV, and EF by Simpson’s method. Multiple regression analysis also showed that longitudinal global strain (beta = −3.335, *p* < 0.001) independently predicted the amount of LVMI regression (Table 3).

**Table 3:** Multiple linear regression analysis of variables for the prediction of left ventricular reverse remodeling

		Estimated beta	standard error	p-value
Model 1	radial strain	1.834	0.839	0.033
	circumferential strain	7.527	3.275	0.026
	longitudinal global strain	−38.444	11.797	0.002
	3D circumferential strain	30.932	12.572	0.017
	3D longitudinal global strain	−4.123	6.884	0.55
	longitudinal global strain: 3D longitudinal global strain	−2.390	0.796	0.004
	3D circumferential strain: 3D longitudinal global strain	2.179	0.840	0.012
Model 2	max velocity of aortic valve	−12.566	4.845	0.012
	longitudinal global strain	−3.335	0.849	<0.001

All strain parameters (6 variables) were calculated by a stepwise multiple linear regression analysis, and several multiple linear regression models were performed with strain parameters or baseline parameters; Model 1, adjusting the stepwise selection as considered with interaction; Model 2, including the main effect term adjusting with baseline parameters such as the age, gender, E velocity, e` velocity, max velocity of aortic valve, ejection fraction by Simpson’s method

All strain parameters were measured by cardiovascular magnetic resonance myocardial tissue tracking

## Discussion

The main findings of the study are that 1) CMR tissue tracking and amount of myocardial fibrosis determined by non-contrast T1 value were significantly correlated; 2) ECV showed a similar correlation pattern with strain by tissue tracking as a non-contrast T1 value; and 3) reverse remodeling as the difference in LVMI between baseline and follow-up Echo was also significantly correlated with myocardial strain by CMR tissue tracking. Global longitudinal strain measured by CMR tissue tracking independently predicted the amount of LVMI regression. In addition, there was some information about the relation between strain and LVMI after AVR in patients with severe AS.

The ability to quantify diffuse myocardial fibrosis in patients noninvasively is of considerable clinical interest, as the pathology of diffuse myocardial fibrosis is reversible with treatment and therefore a potential treatment target [9, 31, 32]. Although widespread and diffuse, the distribution of interstitial myocardial fibrosis in chronic AV disease can be regionally accentuated [33]. CMR can determine the presence, distribution, and quantity of myocardial fibrosis [34]. This study provided further support for the role of CMR tissue tracking as a technique, which demonstrated significant correlation with non-contrast T1 values for assessing myocardial fibrosis.

LV hypertrophy produced by pressure overload in AS is an adaptation that compensates for high intracavitary pressures with the goal of normalizing wall stress and maintaining adequate cardiac output [35, 36]. Persistently elevated systolic wall stress and compromised myocardial perfusion lead to myocyte degeneration and myocardial fibrosis, with a significant relationship between the degree of such morphological alterations and LV function [1, 37, 38]. Azevedo and colleagues also observed that greater degrees of myocardial fibrosis were associated with worse long-term survival after AVR [10]. Removing valvular impedance and wall stress allows ‘reverse remodeling’ of the ventricle and thus improves patient symptoms and prognosis. The beneficial effects of AVR are mainly attributable to a decrease in LV pressure overload, allowing LV mass decrease and regression of hypertrophy [5, 14, 39].

The non-contrast T1 value or ECV could estimate the degree of myocardial fibrosis as anatomical change. On the other hand, the CMR strain parameters by tissue tracking could reflect functional change as deformation parameters. In spite of the low correlation between these parameters, the deformation parameters of CMR strain may be useful outcome parameters without additional T1 mapping sequences. We confirmed that myocardial strain by CMR tissue tracking can independently predict reverse remodeling as LVMI difference, however, the max velocity of AV, mean pressure gradient of AV, and EF as Echo parameters were not significant predictors.

The LV wall is not homogenous and is composed of endocardial, mid-myocardial, and epicardial layers [40]. LV function is determined by the sum of contraction and relaxation in these three layers [41, 42]. As with the progression of AS, the early stage of myocardial fibrosis can develop into subendocardial layers. The results of our study showed that longitudinal global strain as longitudinal function could significantly predict LVMI regression. Only longitudinal function could detect the early progression of myocardial fibrosis. Circumferential strain and radial strain could not detect the early stage of fibrosis progression, because they are related to midwall function. Strain imaging has been demonstrated to be the most appropriate method to evaluate LV myocardial contractility properties and myocardial deformation, as strain may enable a better characterization of subtle changes in LV performance in severe AS patients [43, 44].

CMR tissue tracking is most effective around endocardial borders, most of which are trabeculated [45]. A limitation is the temporal resolution, which may not be able to resolve short-lived phases of cardiac motion in CMR. The frame rate depends on heart rate and various acquisition parameters. Since CMR acquisitions obtain data over several heart beats minor beat-to-beat differences are smoothed out which, in combination with suboptimal temporal resolution, will obscure rapid isovolumic phases and might lead to underestimation of displacement and strain values [46–48].

Tissue tracking was initially developed for 2D images, but the technology can, in principle, be extended to track 3D volumetric regions As a result, some 3D tissue tracking solutions are currently available, although experience with them is still limited [49, 50]. When this extension is feasible, local 3D tissue features may be tracked simultaneously in all directions to derive all deformation parameters. This could theoretically reduce artifacts in deformation such as those that may result from through-plane displacements of 3D structures [51]. 3D acquisitions with comparable resolution in all three orthogonal directions are technically feasible. Although these have yet to be widely implemented, they can be achieved by using relatively long, navigated acquisitions and fast compressed sensing techniques [51].

Analysis of myocardial motion with CMR-tagging is an important tool for the assessment of LV function in several conditions [48], and may help to identify patients before the onset of overt myocardial dysfunction. CMR-tagging has become the reference standard for the evaluation of regional myocardial function [46, 52]. However, CMR-tagging requires specialized tagging sequences and lengthy breath-holds, and the post-processing procedure is laborious and time-consuming [20]. Myocardial tagging may suffer from progressive attenuation of the tag signal during the cardiac cycle [53].

In contrast, CMR tissue tracking requires no acquisitions other than a SSFP sequence, the ‘workhorse’ sequence in CMR. As a further advantage over SPAtial Modulation of Magnetization (SPAMM), in which myocardial tags fade toward the end of diastole, CMR tissue tracking permits measurement of motion and strain throughout the whole cardiac cycle. CMR more consistently provides high-quality imaging with complete LV anatomical regional coverage. CMR tissue tracking does not require additional imaging. Using software, CMR tissue tracking analysis can be performed using routine CMR cine images in less than 10 min [51, 54].

### Limitations

The main limitation of this study is that it is a single center study that included only a small number of patients.

Tissue tagging is still considered the gold standard for strain analysis for research purposes. We could not compare CMR tissue tracking and tissue tagging/SPAMM. In two previous studies, the comparison of CMR tissue tracking and tissue tagging was reported in patients with AS [20, 47]. Until now, there has been no tissue tracking standard reference. Further investigations in different diseases and healthy patients should determine if tissue tracking can serve as a reliable alternative to tagging.

We analyzed the quantification of fibrosis on T1 mapping technique rather than late gadolinium enhancement. T1 mapping is superior at detecting the diffuse fibrosis seen in the pressure overloaded ventricle [8]. Nevertheless, we need to further validate the quantification of fibrosis as tissue tracking in large-scaled population.

Differences in LVMI were measured with the use of 2D Echo. For accurate LVMI measurement, the gold standard is CMR. In this study, baseline LVMI was measured with Echo and CMR. However, only Echo was performed in annual follow-ups. Furthermore, in evaluating LV mass regression in patients with severe AS, we need to assess follow-up CMR after AVR.

## Conclusion

We have shown that longitudinal global strain measured by CMR tissue tracking correlated with the amount of myocardial fibrosis determined by non-contrast T1 values in patients with severe AS. The application of this technique as CMR tissue tracking is feasible in a clinical setting and it has the potential to be used as a simple, non-invasive, non-contrast assessment of myocardial fibrosis using cine sequences. In addition, reverse remodeling as LVMI difference was significantly correlated with myocardial strain by CMR tissue tracking, and longitudinal global strain independently predicted LVMI regression. Further work is needed to determine the role of tissue tracking for monitoring reverse remodeling and to aid risk stratification of AS patients.

## Additional file

Additional file 1:
**Figure S1.** Bland-Altman plot with LVMI values of the baseline Echo and CMR. There was a positive correlation between LVMI values of the baseline Echo and CMR images (*r* = 0.73, *p* < 0.001), and ICC was 0.723 (95% confidence interval: 0.580–0.823, *p* < 0.001). **Figure S2.** The median time duration from AVR to follow-up Echo (median days [interquartile range]: 833[372–1183] days) (PPTX 82 kb)

## Abbreviations

AS: Aortic stenosis
AV: Aortic valve
AVA: Aortic valve area
AVR: Aortic valve replacement
CMR: Cardiovascular magnetic resonance
Echo: Transthoracic echocardiography
EF: Ejection fraction
LV: Left ventricle/left ventricular
LVMI: Left ventricular mass index
