# Supplementary material for: Assessment of reverse remodeling predicted by myocardial deformation on tissue tracking in patients with severe aortic stenosis: a cardiovascular magnetic resonance imaging study
Source: J Cardiovasc Magn Reson. 2017 Oct 23;19:80. doi: 10.1186/s12968-017-0392-0 (PMC5654100; doi:10.1186/s12968-017-0392-0)
Supplement: Additional file 1: — Figure S1. Bland-Altman plot with LVMI values of the baseline Echo and CMR. There was a positive correlation between LVMI values of the baseline Echo and CMR images (r = 0.73, p < 0.001), and ICC was 0.723 (95% confidence interval: 0.580–0.823, p < 0.001). Figure S2. The median time duration from AVR to follow-up Echo (median days [interquartile range]: 833[372–1183] days) (PPTX 82 kb) [file 12968_2017_392_MOESM1_ESM.pptx]

## Slide 1
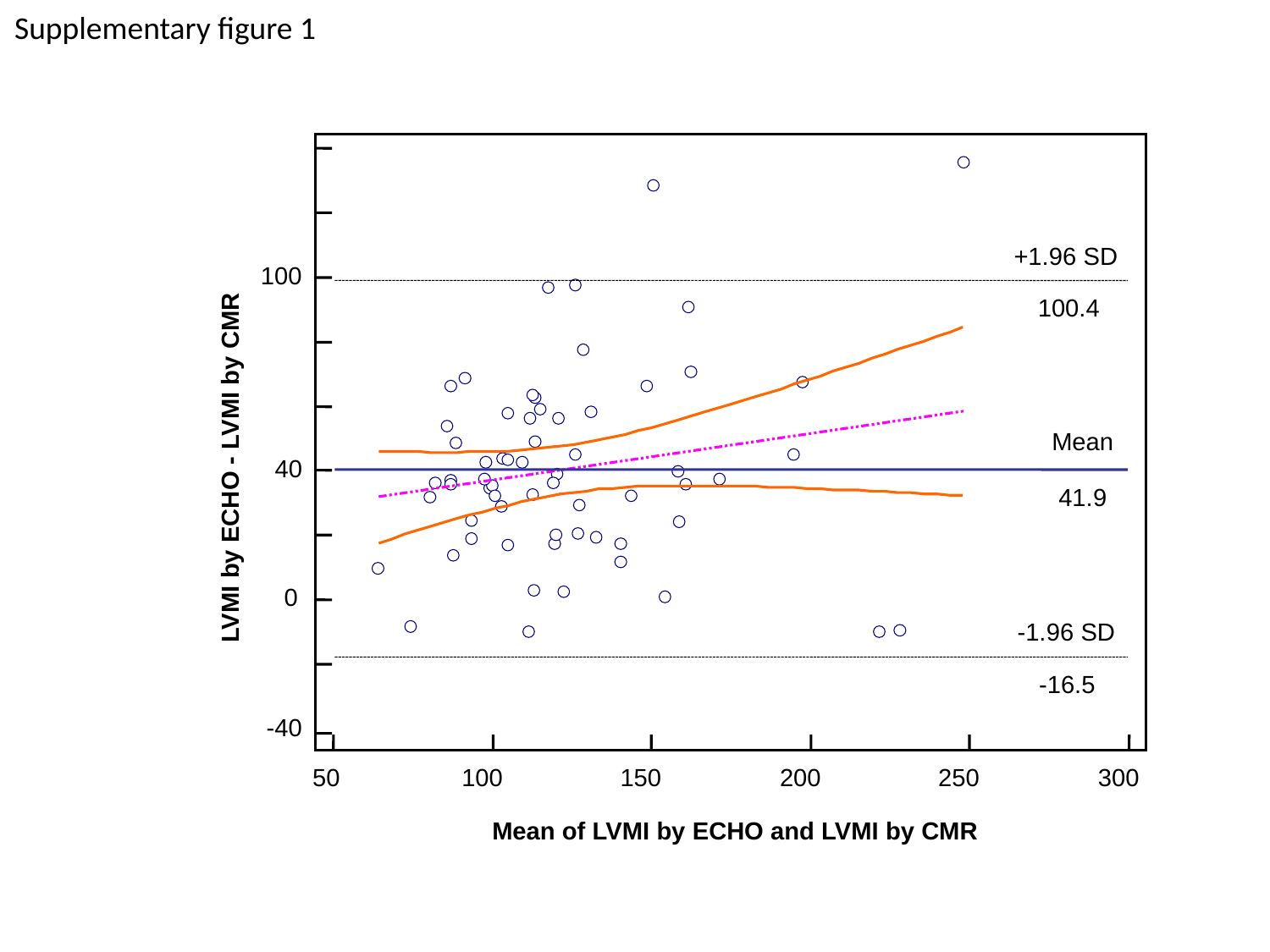

Supplementary figure 1
+1.96 SD
100
100.4
Mean
40
LVMI by ECHO - LVMI by CMR
41.9
0
-1.96 SD
-16.5
-40
50
100
150
200
250
300
Mean of LVMI by ECHO and LVMI by CMR

## Slide 2
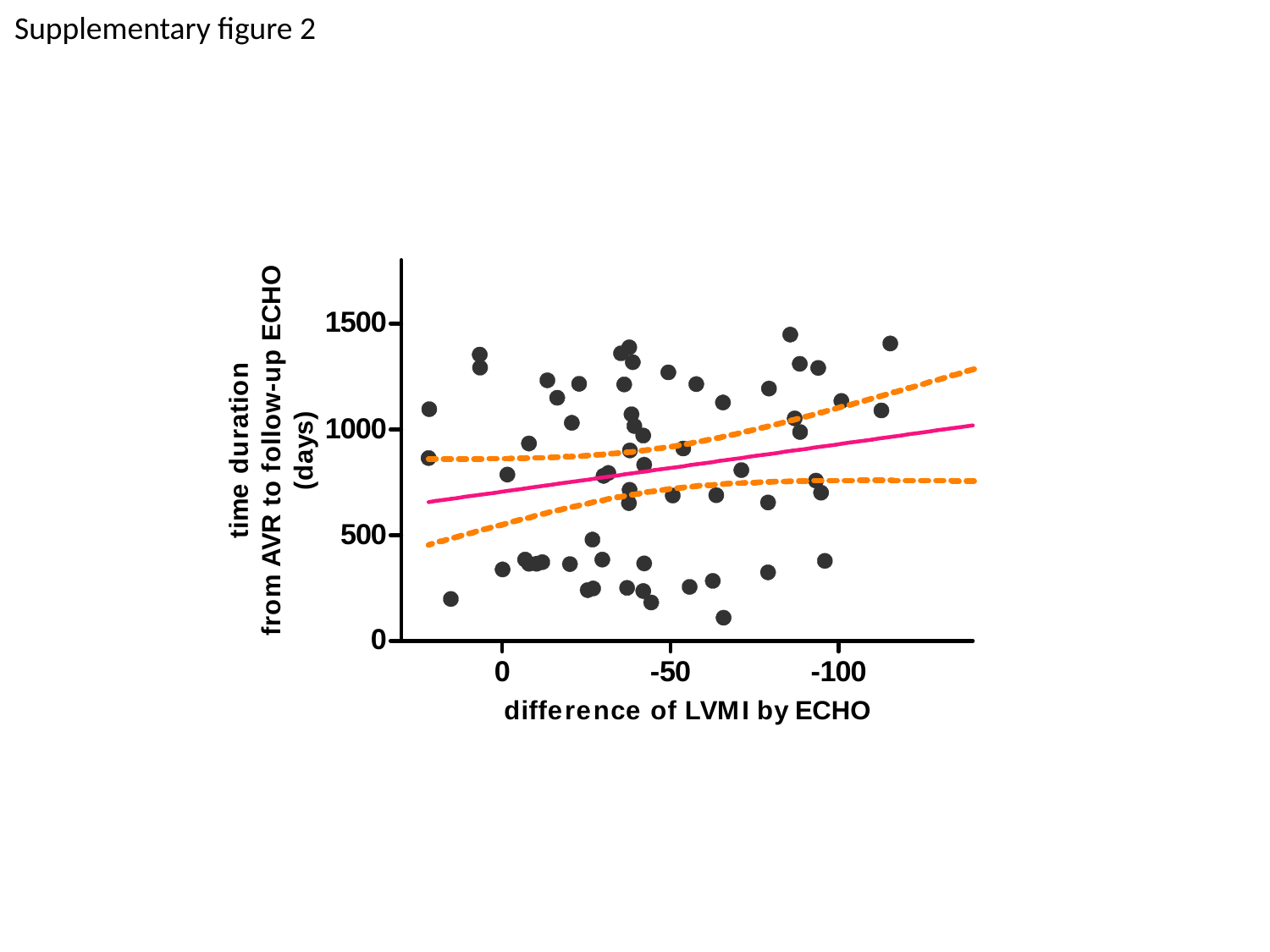

Supplementary figure 2
